# Emotion Regulation and Parental Bonding in Families of Adolescents With Internalizing and Externalizing Symptoms

**DOI:** 10.3389/fpsyg.2018.01493

**Published:** 2018-08-17

**Authors:** Stefania Mannarini, Laura Balottin, Arianna Palmieri, Francesco Carotenuto

**Affiliations:** ^1^Interdepartmental Center for Family Research, Department of Philosophy, Sociology, Education, and Applied Psychology, Section of Applied Psychology, University of Padova, Padova, Italy; ^2^Department of Philosophy, Sociology, Education, and Applied Psychology, Section of Applied Psychology, University of Padova, Padova, Italy

**Keywords:** adolescence, psychopathology, family, intergenerational transmission, alexithymia

## Abstract

Parental bonding and emotional regulation, while important to explain difficulties that may arise in child development, have mainly been studied at an individual level. The present study aims to examine alexithymia and parental bonding in families of adolescents with psychiatric disorders through different generations. The sample included a total of 102 adolescent patients with psychiatric disorders and their parents. In order to take a family level approach, a Latent Class Analysis was used to identify the latent relationships among alexithymia (Toronto Alexithymia Scale), perceived parental bonding (Parental Bonding Instrument) and the presence of adolescent internalizing or externalizing psychiatric symptoms (Youth Self-Report). Families of internalizing and externalizing adolescents present different and specific patterns of emotional regulation and parenting. High levels of adolescent alexithymia, along with a neglectful parenting style perceived by the adolescent and the father as well, characterized the families of patients with internalizing symptoms. On the other hand, in the families with externalizing adolescents, it was mainly the mother to remember an affectionless control parental style. These results suggest the existence of an intergenerational transmission of specific parental bonding, which may influence the emotional regulation and therefore the manifestation of psychiatric symptoms.

## Introduction

The family is one of the main living contexts that influence the development of adolescents: the answer offered by the environment, in particular within the parent–child relationship, challenged by and involved in the inner conflicts of the adolescent, plays a crucial role, in the child’s development (Jeammet, 2004, 2010). The parenting, that is a set of behaviors that relates to the ability to protect the child and support its development, requires a variety of skills and sensitivity to the different needs of the child (Bornstein, 2002). Within the family environment adolescence is a crucial period for the development of disorders that can influence adult life too (Reef et al., 2010; Nobile et al., 2013; Ormel et al., 2015). Research on parenting styles and psychosocial well-being conducted on the general population showed that a parent–adolescent relationship characterized by high levels of care and warmth is associated with minor levels of internalizing and externalizing problems in the offspring; conversely parental practices characterized by low care and high overprotection can make the offspring vulnerable to psychopathology (Rohner and Britner, 2002; Andersson and Eisemann, 2003; Avagianou and Zafiropoulou, 2008; Boričević Maršanić et al., 2014; Freeze et al., 2014; Sarajlić Vuković et al., 2015).

The perceived parental bonding and the representations of attachment bonds are in fact closely related to the emotional development of the child and with her/his possibility to learn more or less functional ways for regulating inner affects and emotions (Tasca et al., 2009). Among the deficits in emotion regulation, alexithymia is considered a processing disorder of affects that interferes with self-regulation processes and symbolization of emotions, characterized by a series of difficulties in: identifying, describing and interpreting subjective and others’ feelings; distinguishing emotional states from physiological perceptions; identifying the causes of one’s own emotions; using language to express feelings, which result in a tendency to replace words with actions (externally oriented way of thinking) (Taylor et al., 1997). Current research suggests that alexithymia is one of the predisposing factors in many psychiatric disorders, either with internalizing or externalizing symptoms, during preadolescence, adolescence and adulthood too (Honkalampi et al., 2009; Manninen et al., 2011; Rieffe and De Rooij, 2012; Di Trani et al., 2013; Mannarini et al., 2016; Schimmenti et al., 2017). Internalizing disorders, such as anxiety, depression, somatic disorders and eating behavior problems, are a specific type of emotional and behavioral problems, probably based on an inappropriate or maladaptive control or regulation of emotional and cognitive states, which are sometimes difficult to identify through external observations (Merrell, 2008). Whereas in the externalizing disorders, behavioral problems are directed to the outside and negatively affecting people and the surrounding environment: examples can be disobedience, aggression, impulsive and violent behaviors, contesting, non-respect of rules and property destruction (Manninen et al., 2011).

Moreover research in both non-clinical and clinical samples, synthetized in a comprehensive meta-analysis (Thorberg et al., 2011), suggest a negative association between paternal and maternal care and alexithymia, while paternal and maternal overprotection were significantly and positively associated with the alexithymic deficit. These effects appeared to be more marked in clinical samples (Kooiman et al., 2004; De Panfilis et al., 2008; Hsu et al., 2013). These researches, although conducted mainly on adult samples, promote the hypothesis of a deep connection between parental bonding -considered among the most important environmental factors- and potential deficits in emotion regulation, such as alexithymia. Various models have been proposed to explain the etiology of emotional dysregulation, among which the hypothesis that a perceived emotional distance on the parents’ part or a discontinuity experienced in the relationship by the child can negatively affects the latter’s ability to regulate his own emotions (Lumley et al., 1996; Taylor et al., 1997).

As a consequence of this hypothesis, literature suggests the existence of a potential intergenerational transmission of the difficulty in emotional regulation from parents to their children (Lumley et al., 1996; Fukunishi and Paris, 2001; Guttman and Laporte, 2002; Kliewer et al., 2016). Nevertheless alexithymia along with parental bonding have been usually studied only at the individual level both in the general population and in psychiatric patients, including recently adolescents too. To our knowledge no studies have examined these constructs at a family level, looking for a relationship between alexithymia and parental bonding trough different generations from parents to adolescent offspring with psychiatric disorders, which was the primary aim of the present study.

Few studies have tried to investigate the intergenerational transmission of alexithymia solely from parents to children, in particular in samples of pre-adolescents and adolescents with psychiatric diagnoses. These pioneering studies suggested that parental emotional dysfunctions both from mother and father provide similar problems to their children (Guttman and Laporte, 2002; Balottin et al., 2014; Duclos et al., 2014; Gatta et al., 2017). Concerning the parental bonding solely, although the attachment theoretical model indicates the mutual influence of parental styles and of attachment throughout generations (grandparents, parents and children), very few studies examined the perception of parental bonding on the part of adolescent patients with psychiatric diagnoses and their parents. These studies aimed to assess a possible continuity and influence of different attachment styles between generations (parents to their own parents – children to their parents) within the families of adolescents with specific psychopathologies, such as eating disorders and borderline personality disorder (Canetti et al., 2008; Infurna et al., 2016; Balottin et al., 2017). The findings showed that a careless and overcontrolling parental style, perceived in particular by the father, characterized the families of adolescent patients with anorexia nervosa (Balottin et al., 2017); while adolescents with borderline personality disorder experience both their mother and father as lacking in care and overcontrolling, and similarly their parents remember their own parents as non-affectionate (Infurna et al., 2016).

Literature indicate on the whole that there is a complex non-linear relationship between the emotional functioning of parents and their children and between their recalled parental bonding styles and those of their offspring, which may differ in different psychiatric disorders and deserve to be explored more in depth. The present study thus aims therefore to represent a contribution to the still lacking literature on the intergenerational patterns of parental bonding and emotion regulation in adolescents with psychiatric disorders. In order to take a family level approach, the perceived parental bonding were assessed for what concerns both the mother and the father of the adolescents, and also parental perceptions of their family of origin were considered. Parental bonding was therefore explored trough two generations (parents to their own parents – children to their parents). Potential deficits in emotion regulation were also investigated in adolescents and in parents as well. A Latent Class Analysis approach was adopted to identify at a family level the latent relationship among alexithymia, perceived parental bonding and the presence of internalizing or externalizing psychiatric symptoms in the adolescents. The main aim of the study was therefore evaluating whether the families of adolescents with internalizing/ externalizing symptoms exhibit different specific patterns of parental bonding and emotion regulation at a triadic level.

## Materials and Methods

### Participants

The sample (*N* = 102) included adolescents with psychiatric disorders and parents, consecutively referred to a northern Italian psychiatric out-patient services specifically addressed to children and families from May to December 2016. The clinical group consists of 34 adolescents (14 males and 20 females) with a mean age of 15.44 years (*SD* = 1.76), pre-adolescents (52.9%) and adolescents (47.1%) ranging from 12 to 18 years. 25 patients had at least one sibling, while 9 were only children. The 34 fathers’ age ranged from 39 to 63 years (*M* = 50,45; *sd* = 5,44), while the 34 mothers’ age ranged from 38 to 59 years (*M* = 46,59; *ds* = 4,43). The 18% of the parents were divorced, while the 82% were married or cohabiting. **Table 1** shows the socio-economic and clinical characteristics of the adolescent patients, including their psychiatric diagnoses.

**Table 1:** Patient’s socio-demographic and clinical characteristics.

SES	Patients (%)
Low/Medium-low	41.2
Medium	26.5
Medium-high/High	32.4

**ICD-10 Diagnoses**	**Patients (%)**

Emotional and behavioral disorders of adolescence	28.4
Personality and behavioral disorders	21.6
Neurotic, stress-related or somatoform disorders	30.4
Mood (affective) disorders	19.6

*SES, socio-economic status, calculated using the Hollingshead’s Four Factor Index of Social Status (SES). ICD-10 Diagnoses, diagnostic groupings based on ICD-10 codes.*

### Procedure

All adolescents and participants’ parents gave their written informed consent to participate in the study. The study was conducted in accordance with the national and institutional code of good ethical practice. The local ethics committee for psychological research (area 17) of the University of Padova (2015) approved the research protocol.

The participants adhered to the research after the first consultations with psychologists or psychiatrists. After being informed verbally about the possibility to join the project and the modalities of the participation, parents were asked to read and sign an informed consent, in which the study procedure and the privacy statements (Italian D.lgs. 196/2003) were presented. The parents of adolescents with primary neurological diseases or particularly serious psychological disorders were excluded. The ICD-10 diagnoses, along with the Youth Self-Report scores totalized by patients (and in particular the DSM-IV oriented scales), were used to define the prevalent psychiatric disorder, in order to distinguish two categories of patients: internalizing or externalizing.

### Measures

#### The Youth Self-Report (YSR 11-18)

Youth self-report (Achembach and Rescorla, 2001) is a self-report questionnaire for preadolescents and adolescents aged from 11 to 18 years. It assesses the areas of competences and social problems (activities, social functioning, and school performance), investigating the overall functioning of the adolescent. Moreover specific emotional and behavioral problems can be assessed and categorized as “normal,” “borderline,” or “clinical.” The 8 empirically based syndromes are anxious/depressed, withdrawn/depressed, somatic complaints, social problems, thought problems, attention problems, delinquent behavior, and aggressive behaviors. Additionally these scales are grouped into Internalizing problems (which is the sum of the first three sub-scales) and Externalizing problems (the sum of the last two sub-scales). The instrument provides also a total score for problems including both Internalizing and Externalizing problems. For these three scales the T score ranges for the cut offs are as follows: normal = score ≤ 59; borderline = score from 60 to 63; clinical = score ≥ 64. Moreover six syndromic scales based on DSM-IV were created to assess somatization, anxiety disorders, affective disorders, ADHD, oppositional defiant disorder and conduct disorder. This instrument has been used in several studies all over the world (Ivanova et al., 2007) and in Italy too (Frigerio et al., 2009), showing always a good validity and reliability (α > 0.65).

#### The Parental Bonding Instrument (PBI)

The PBI (Parker et al., 1979) is a self-report questionnaire aimed at measuring the perceived parenting style during infancy and adolescence. The instrument consists of 2 parts which concerns the perception of the mother’s and the father’s parenting style: both parts are composed by 25 identical items. The perceived parenting style is measured on the two dimensions of care and overprotection. The care dimension concerns the sense of warmth and perceived emotional involvement while overprotection explores the perceived parental control. For paternal bonding the cut off are: low care = score ≤ 24.0, high care = score > 24.0; low overprotection = score ≤ 12.5, high overprotection = score > 12.5. For maternal one the cut off are: low care = score ≤ 27.0, high care = score > 27.0; low overprotection = score ≤ 13.5, high overprotection = score > 13.5. Four parenting styles were obtained by crossing these two dimensions: “optimal parenting (high care – low overprotection) neglectful parenting (low care – low overprotection), affectionate constraint (high care – high overprotection), and finally affectionless control” (low care – high overprotection). The instrument has demonstrated good reliability and stability and has been validated in different countries, among whom Italy (Favaretto et al., 2001).

#### The Toronto Alexithymia Scale (TAS-20)

The TAS-20 (Bagby et al., 1994) is the most used self-report questionnaire for measuring alexithymia. It consists of 20 items rated on a 5-point Likert scale. It provides a total score and measures the three main dimension of alexithymia: difficulties identifying feelings (F1); difficulties describing feelings (F2); and externally oriented thinking (F3). The total score identifies three categories: no alexithymia = score < 52, borderline = score from 52 to 60 and alexithymia = score > 61. The instrument has been widely validated, and demonstrated good reliability and stability also in its Italian version (Bressi et al., 1996).

### Analyses

As a first approach to the data, in order to examine at a manifest level the PBI and TAS-20 responses, before applying the Latent Class Analysis, the parental care and overprotection variables, as well as the alexithymia variable, were analyzed using frequencies and means in the families of adolescents with internalizing/externalizing psychiatric symptoms.

### The Latent Class Analysis

The latent class analysis (LCA) procedure (e.g., Hagenaars and McCutcheon, 2006) was selected to locate a latent categorical variable. This is useful for identifying a hypothetical construct that takes into account the covariance between the observed variables. The LCA presupposes that the latent variable is categorical and consists of a number of classes that describe the presence or absence of the attributes of the latent variable. Each of these classes should represent a “profile” in relation to the specific variables present in the study: family roles, typical of adolescents’ families with internalizing (group 1) vs. externalizing (group 2) psychiatric symptoms, care, overprotection and alexithymia. In this context a one latent variable with two classes representing the two family groups was hypothesized. Equal hypotheses were formulated separately for both maternal and paternal bonding. It was supposed that the two latent class structure were different in relation to paternal and maternal bonding. Nevertheless, given the exploratory nature of the modeling approach used in this study, no specific hypothesis on how the structure of paternal and maternal bond can differ has been advanced. The manifest variables included in the LCA model were the family role (adolescent, mother, and father), the families of adolescent with psychiatric symptoms (two groups: internalizing vs. externalizing), the PBI variable care (two levels: low care and high care), the PBI variable overprotection (two levels: low overprotection and high overprotection) and the variable alexithymia (two levels: non-clinical scores in the normal range vs. borderline/clinical level of alexithymia).

The measurement model of the LCA has been widely used both in the psychological and medical literature to model the possible relationships among the variables with the aim of identifying the underlying structure characterized by one or more categorical latent variables so as to draw a profile of the people who fall in one class or another (Mannarini, 2009, 2010; Mannarini and Boffo, 2013, 2014, 2015; Mannarini et al., 2018). The model fitting the data was evaluated by looking at the Akaike information criterion (AIC) and the Bayesian information criterion (BIC) considering also the Chi square and the Likelihood Ratio statistics. The model has a good validity in both statistics with a probability higher than *p* = 0.05. The LCA analysis was executed with LEM program (Vermunt, 1997).

## Results

### Description of Families of Adolescents With Internalizing and Externalizing Symptoms

The percentage frequencies of adolescents, fathers and mothers in relation to alexithymia (TAS-20), care and overprotection perceived in the parental bonding (PBI) are reported in **Table 2**. No distinction between internalizing and externalizing families is made in this context. Taking into consideration the highest percentage values, it is worth noticing adolescents and fathers both perceive low care on the fathers’ part and low overprotection on the mothers’ part (PBI). Mean alexithymia scores (TAS-20) are 57.2 (*sd* = 10.4) for the adolescents, 47.1 (*sd* = 11.6) for the fathers and 44.2 (*sd* = 11.4) for the mothers.

**Table 2:** Alexithymia (TAS-20), Care and Overprotection levels (PBI Scales) in adolescents and parents: percentage frequency distributions.

TAS-20	Adolescent (%)		Father (%)		Mother (%)	
Non-clinical	26.2		67.6		73.5	
Borderline	26.2		11.8		14.7	
Clinical	47.6		20.6		11.8	

**PBI**	**Adolescent (%) Paternal**	**Maternal**	**Father (%) Paternal**	**Maternal**	**Mother (%) Paternal**	**Maternal**

Care						
Low	61.8	47.1	67.6	58.8	58.8	58.8
High	38.2	52.9	32.4	41.2	41.2	41.2
Overprotection						
Low	58.8	61.8	58.8	79.4	47.1	61.8
High	41.2	38.2	41.2	20.6	52.9	38.2

### Latent Class Analysis

The LCA analysis identified two-latent-class structures separately for paternal and maternal bonding, each one represented by two classes. The indices fitting the data were Chi *square* = 15.11, *df* = 22, *p* = 0.86, L_2_ = 19.10, *df* = 22, *p* = 0.64, AIC (L_2_) = -24.90, BIC (L_2_) = -82.65 for paternal bonding, and Chi *square* = 25.36, *df* = 22, *p* = 0.28, L_2_ = 27.98, *df* = 22, *p* = 0.18, AIC (L_2_) = -16.01, BIC (L_2_) = -73.77 for maternal bonding.

Identify the latent structures, the probability parameter estimates for each latent class showed their peculiar characteristics (see **Table 3**). For both paternal and maternal bonding, Class 1 and Class 2 presented probabilities, in the range 0.37 -0.63. Each class evidenced different probability parameter estimates: probability values significant for the latent class interpretation were highlighted in bold in **Table 3**.

**Table 3:** Two-classes latent class analyses: parental bonding and maternal bonding.

Latent class		Paternal bonding		Maternal bonding	
		
		Class 1 (0.52)	Class 2 (0.48)	Class 1 (0.63)	Class 2 (0.37)
Group	Internalizing	0.34	**0.67**	**0.72**	0.13
	Externalizing	**0.66**	0.33	0.28	**0.87**
Family role	Adolescent	0.19	**0.49**	0.30	0.39
	Father	0.34	0.33	**0.44**	0.16
	Mother	**0.47**	0.18	0.27	**0.45**
Care	Low	**0.64**	**0.61**	**0.67**	0.35
	High	0.36	0.39	0.33	**0.65**
Overprotection	Low	0.48	**0.62**	**0.65**	**0.72**
	High	**0.52**	0.38	0.35	0.28
Alexithymia	Low	**0.87**	0.41	**0.57**	**0.77**
	High	0.13	**0.59**	0.43	0.23

*Paternal bonding and Maternal bonding, Latent variables; Group, Family Roles, Care and Overprotection (PBI) and Alexithymia (TAS-20), Manifest variables. Substantial probability values for the interpretation are evidenced in bold.*

Concerning *paternal bonding*, Class 1 is represented by the families of patients with externalizing symptoms (0.66). Here too the paternal bonding differentiated the mothers (0.47), characterized by low care (0.64) and high protection (0.52) and a low level of alexithymia (0.87). Class 2 instead is represented by the families of patients with internalizing symptoms (0.67), where the paternal bonding is specific in particular for the adolescent (0.49), characterized by low care (0.61) and low protection (0.62), along with a high level of alexithymia (0.59).

Concerning *maternal bonding*, Class 1 is represented by the families of patients with internalizing symptoms (0.72). In these families, the maternal bonding appeared to differentiate in particular the fathers’ perception (0.44), characterized by low care (0.67) and low protection (0.65), along with a low level of alexithymia (0.57). Class 2 is represented by the families of patients with externalizing symptoms (0.87) and in this case the maternal bonding appeared to differentiate in particular the mothers (0.45), characterized by high care (0.65) and low protection (0.72), and a low level of alexithymia (0.77).

Considering the two groups of families, there are similarities and differences between them. In families of patients with internalizing symptoms, the adolescents perceive a weak or absent bonding with the father, which is accompanied by a marked alexithymia. Moreover the adolescents’ paternal bonding is distinguished by specificities concerning the relationship revoked with his mother. In these families both maternal bonding as perceived by the father and paternal one as perceived by the adolescent are characterized by low care and low protection. The only difference between the adolescent and the father concerned alexithymia.

For families of patients with externalizing symptoms, the parental bonding revoked by the mothers is characterized by specificities with both parents. Paternal bonding was characterized by low care and high protection, i.e., an affectionless-control parental style, while the maternal one by high care and low overprotection which corresponds to an optimal parental style.

The results of the latent analyses in large part were confirmed by the results of the analysis of the associations among the variables of the LCA model, in particular both paternal and maternal bonding latent variables appeared to be associated to the kind of psychiatric symptoms (internalizing vs. externalizing, *p* < 0.001). Considering the family roles, a significant association was found with paternal and maternal bonding (*p* < 0.001). As regards the PBI variables again for both paternal and maternal bonding the association with care and overprotection was significant (*p* < 0.001). Maternal bonding latent variable was also strongly associated separately with care (*p* < 0.001) and overprotection (*p* < 0.001). The relation between the latent variable and alexithymia was evidenced only as far as maternal bonding is concerned (*p* < 0.001).

## Discussion

Comparing the families of adolescents with internalizing symptoms and those with externalizing ones, specific pattern of emotion regulation and parental bonding were identified. The families of patients with internalizing symptoms appeared to be characterized specifically by a difficulty on the adolescent’s part in identifying, describing and thinking on emotional experiences, due to an elevated level of alexithymia. This characteristic was found to be linked to the perception of a careless and affectionless parenting on the father’s part, characterized by a weak or absent bonding. Moreover in these families also the fathers themselves, differently from the mothers, reported to have perceived weak or absent bonding with their own mothers. The paternal line emerged therefore to be very significant in the families of patients with internalizing symptoms, favoring the hypothesis of a potential intergenerational transmission of the parental bonding from father to offspring.

These results confirm and expand previous literature on the issue, since recent studies (Rohner and Britner, 2002; Avagianou and Zafiropoulou, 2008; Boričević Maršanić et al., 2014) has shown that a parenting style characterized by low emotional warmth and empathy along with rejection is associated with internalizing symptoms in both clinical and non-clinical adolescents in different countries. Moreover, other studies suggest that also alexithymia is a predisposing factor for both internalizing psychopathology in preadolescent and adolescent age (Ciarrochi et al., 2008; Rieffe and De Rooij, 2012). Some studies have investigated therefore the relationship between parental bonding and alexithymia; however, most of these studies have been conducted on non-clinical and clinical samples of adults, while few studies have investigated this connection in clinical samples of adolescents with psychiatric disorders, suggesting that a lack of both paternal and maternal care was associated with high levels of alexithymia in children (Kooiman et al., 2004; Thorberg et al., 2011; Duclos et al., 2014; Gatta et al., 2017).

Assuming an intergenerational perspective in interpreting our results, it can be hypothesized that the memories of the adolescents’ fathers, who experienced a relationship with their own mothers characterized by lack of affection, care and protection, could led them to transfer this same kind of parenting behaviors toward their offspring. Within the families of patients with internalizing symptoms, the adolescents’ relationships with their fathers appeared indeed problematic, since they perceive to be rejected and not protected just as their fathers felt by their own mother. This can lead the adolescents to feel unappreciated and to wonder why they are not loved by the parental figure, experiencing feelings of anxiety, depression and frustration. These ambivalent affections toward themself and their fathers may result in difficulties in identifying, describing or thinking the emotions. The link emerged in our sample, among parenting style, alexithymia and internalizing symptoms lead to support the hypothesis that alexithymia is an important factor in explaining the association between perceived dysfunctional parenting styles and higher risk for internalizing symptoms.

Moreover our results clearly showed that not only the perceived relationship with the mother but also with the father can deeply influence the ability to think about emotions and affections and therefore the manifestation of internalizing symptoms. In a previous study, conducted on a sample of adolescents with psychiatric disorders, Gatta et al. (2017) found that the adolescents who perceived their fathers as neglectful showed greater emotional difficulties, and this emotional deficits could contribute to their psychiatric outcome. This study shows that, in families of internalizing adolescents, also the fathers themselves recall to have experienced an analogous parenting style during their own infancy. Having experienced a negligent parenting style through the generations could probably contribute to the existence of an internalizing pathology in children by affecting the ability of the adolescent to identify, describe and express emotions.

Differently from what previously described, the adolescents and the fathers didn’t contribute significantly in characterizing the families of patients with externalizing symptoms. It was in fact essentially the mothers to recall and perceive their own fathers as over controlling but scarcely affective. Literature confirms this style of parenting to be associated with externalizing symptoms: parenting styles characterized mainly by strict discipline, high severity, limitation of autonomy, overprotection and rejection are associated with a variety of psychiatric disorders in adulthood, suggesting that these parenting practices can make the offspring vulnerable to externalizing psychopathology (Rohner and Britner, 2002; Boričević Maršanić et al., 2014; Freeze et al., 2014; Sarajlić Vuković et al., 2015). In particular, Buschgens et al. (2010) found in a sample of pre-adolescent who had experienced a lack of emotional warmth and a high overprotection by parents, that they were described by parents and teachers as more aggressive and prone to criminal acts, as well as more hyperactive.

But what is surprising in our results is that the perception of a controlling parental style is predominantly maternal rather than adolescent. In line with our results, a study of Infurna et al. (2016) on adolescent patients with borderline personality disorder, find evidence of transgenerational effects of parental bonding: the parents’ experience of a parenting style characterized by low care and high overprotection affect in fact their own parental practices later with their adolescent offspring. From an intergenerational point of view, we can hypothesize that the mothers’ perception of having experienced during childhood a relationship with a controlling paternal figure without emotional warmth may also lead them to experience more difficulties with the offspring in particular during adolescence, which is a period of life often characterized by a struggle for autonomy. The perception of the maternal struggle with authority issues may also lead the adolescent offspring to act directly the emotions without thinking and in a more aggressive way, perhaps also to attract parental attention (Berastegui et al., 2012).

A limit of the study consists in the assessment of alexithymia in adolescents by using the TAS-20, since the instrument, conceived for an adult population, may present some difficulties for the younger patients. Despite the majority of the participants were 15 or older, literature (Rieffe et al., 2006; Säkkinen et al., 2007; Zimmermann et al., 2007; Di Trani et al., 2018) highlighted several critical issues in measuring alexithymia in adolescents with adult self-reports. The differences found in the average level of alexithymia across different ages suggest caution in interpreting the results of the present study. There are indeed no Italian validations in adolescent samples for the TAS-20, differently from the children version, firstly created by Rieffe et al. (2006) and validated in an Italian sample by Di Trani et al. (2018). Despite using the TAS-20 and the PBI scales in the parents as well as in the adolescents allowed parents’ responses and their offspring’s ones to be comparable, subsequent studies would benefit from a multi-informant evaluation. This kind of evaluation would be more precise, especially in detecting emotional difficulties, which young people might not always be completely aware of (Taylor and Bagby, 2004).

Although other limitations of this study must be underlined, such as the lack of a control group to compare clinical subjects and the limited number of participants, our study represents a contribution to the scientific literature dealing with the intergenerational transmission of parental bonding and emotion regulation in adolescents with psychiatric disorders. The study identified the crucial role of both mothers and fathers in the emotional and psychic development of the adolescent (Shelton and Harold, 2008; Gruhn et al., 2016). Parents’ perceptions of having experienced maladaptive parenting styles (either neglectful or affectionless control) seem to have repercussions on the subsequent generation, manifesting internalizing or externalizing symptoms. To our knowledge, this is the first study, conducted in a sample of clinical preadolescents and adolescents, to consider simultaneously the maternal, paternal and adolescent parenting styles and style of emotional regulation, i.e., parental bonding and alexithymia through two generations.

Our results underline similarities and discordances between families of patients with internalizing and externalizing symptoms. In both internalizing and externalizing families it not only the child’s perception of parenting styles but also the perception of the parents regarding their own parents appeared to be problematic. However, important differences emerged concerning the paternal and maternal role. In families of adolescents with internalizing symptoms, the paternal figure, perceiving a negligent parenting style, plays a major role, while in externalizing families it was the mother to perceive a parenting style characterized by lack of affection and hypercontrol by the father. Moreover, a difficulty in identifying and expressing emotions has emerged specifically in families with children with internalizing symptoms, while boys with externalizing symptomatology, being more prone to the direct passage to the act, probably show a minor awareness of their emotional difficulties.

As a clinical implication, exploring the maternal and paternal parenting styles could help to identify the risk of developing internalizing or externalizing symptoms in the adolescents, with a positive impact on the possibilities of prevention. Also in the case of an adolescent internalizing or externalizing structured psychopathology, evaluating the complex dynamics of the family triad along the different generations can be useful. This would help parents to become more aware of both their parenting behaviors and those of their parents and to prevent dysfunctional parenting behaviors to be transmitted from generation to generation and coercively repeated in a dysfunctional circle. Comprehensive clinical evaluation of adolescent psychopathology, as well as future research, should therefore focus not only on the perception of parenting styles of the child toward the parents but also on the parental perceptions, trying to elucidate the differences among different psychiatric outcomes, such as internalizing versus externalizing symptoms. Future research that will study the complex dynamics linked to maladaptive intergenerational transmission may help clinicians to structure better therapies not only based on the individual patients’ needs but also on family’ needs. In fact, the involvement of parents in the treatment of their children with psychiatric disorders could benefit not only the patients’ well-being but also the whole family as well.

## Author Contributions

SM, LB, and FC wrote the manuscript. SM, LB, and AP contributed to study design. FC contributed to data collection. SM performed analyses and contributed to data interpretation. All the authors approved the final manuscript and agreed to be accountable for all the aspects of the study.

## Conflict of Interest Statement

The authors declare that the research was conducted in the absence of any commercial or financial relationships that could be construed as a potential conflict of interest.
